# Teacher-learner interaction quantifies scaffolding behaviour in imitation learning

**DOI:** 10.1038/s41598-019-44049-x

**Published:** 2019-05-17

**Authors:** Shuntaro Okazaki, Yoshihiro Muraoka, Rieko Osu

**Affiliations:** Faculty of Human Sciences, Waseda University, Saitama, Japan

**Keywords:** Human behaviour, Time series

## Abstract

Teachers often believe that they take into account learners’ ongoing learning progress in their teaching. Can behavioural data support this belief? To address this question, we investigated the interactive behavioural coordination between teachers and learners during imitation learning to solve a puzzle. The teacher manually demonstrated the puzzle solution to a learner who immediately imitated and learned it. Manual movements of teachers and learners were analysed using a bivariate autoregressive model. To identify bidirectional information exchange and information shared between the two agents, we calculated causality and noise covariance from the model. Information transfer observed from teacher to learner in the lateral component of their motion indicated imitation of the spatial information of the puzzle solution. Information transfer from learner to teacher in the vertical component of their motion indicated the monitoring process through which teachers adjust their timing of demonstration to the learner’s progress. The shared information in the lateral component increased as learning progressed, indicating the knowledge was shared between the two agents. Our findings demonstrated that the teacher interactively engaged in and contingently supported (i.e. scaffolded) imitation. We thus provide a behavioural signature of the teacher’s intention to promote learning indispensable for understanding the nature of teaching.

## Introduction

Teaching is a fundamental human activity to transfer and accumulate knowledge and skills among people and their descendants, ultimately fostering their culture^1,2^. Teaching, by definition, involves at least two agents: teacher and learner. This makes learning in the context of teaching different from learning without a teacher^3^. Learners vary in their level of understanding and there is no perfect teacher for all students. The teacher has to adapt her/his teaching behaviour to the learner’s variation^4^. Humans, as well as some non-human animals, can also modify their behaviour and promote learning^5,6^. However, even now, there has been much debate about whether teaching is human-unique or not^7,8^. Some researchers have accepted the teaching behaviour in non-human animals based on the operational definition that teaching can be identified by observable behaviour^9–12^, whereas other researchers still emphasize that teaching is unique to humans from the cognitive perspective that it requires complex cognitive processes in the brain^1,13^. To solve the controversial issue of what teaching is, it is widely agreed that we need to determine whether or not the teacher intends to promote learning^4,7,14,15^. However, teaching as an act of intentional communication has been challenged in the case of absence of language^15^. Here we address the question of how this intention can be identified from the teaching behaviour itself, rather than through teachers’ verbally communicated intent. Behavioural signs of teaching intent will provide a valuable opportunity to investigate the nature of teaching in non-human animals, as well as in humans.

We consider the scaffolding behaviour during teaching to provide an opportunity to quantify teaching intention. Scaffolding, a metaphor first adopted by Bruner^16^ and Wood *et al*.^17^, originally indicates the adult’s assistance that is controlled to fill the child- or novice-specific gap between what s/he recognizes and what s/he actually does in a task or in achieving a goal. More generally, scaffolding is defined as a teacher’s various supports to promote learning by contingently responding to the learner’s need^17^. While most studies of scaffolding are descriptive and focus on teaching with verbal instruction, quantifying the scaffolding behaviour that fulfils the three key characteristics recently proposed^18^, would allow us to identify teaching intention from the teaching behaviour. First is *contingency*: the teacher’s support is interactively tailored or adjusted to the current level of the learner’s performance; second is *fading* of this support; and, third is *transfer of responsibility*, i.e., the learner’s contribution to the task accomplishment increases together with the fading of support. In other words, scaffolding is a truly interactive process that occurs between teacher and learner with both participating actively in the process^18^. Some quantitative studies have demonstrated non-verbal scaffolding such as exaggerated and slowed demonstration, or physical guidance when a human adult teaches a specific action to an infant or a robot by imitation^19–21^. However, it is unclear whether these behaviours for infants and robots fulfilled the three characteristics of scaffolding because it might be a unidirectional support based on teachers’ *a priori* information about the learners (age or appearance). To tackle this issue, it is necessary to investigate the dynamic process of information exchange between the teacher and the learner to quantify the scaffolding process^1^. Kostrubiec and colleagues have recently reported empirical evidence of bidirectional information flow between a computer-driven virtual teacher and human learner using a sophisticated experimental setup^22^. However, it remains elusive how a human teacher actually interacts with a learner to promote her/his learning because the virtual teacher adjusts its behaviour in a designated manner. The authors could not demonstrate clear evidence of contingency and fading support in the information transfer.

Imitation learning in the context of teaching by demonstration can be a good example of such teacher-learner interaction. As described, scaffolding behaviour has often been observed in teachers’ demonstrations for imitation^19–21^. Teacher involvement in imitation learning has been recently addressed in the neuroscience literature^23^. Investigating teacher-learner interaction during imitation learning is a straightforward solution to quantify scaffolding behaviour and reveal its mechanisms.

**Figure 1:**
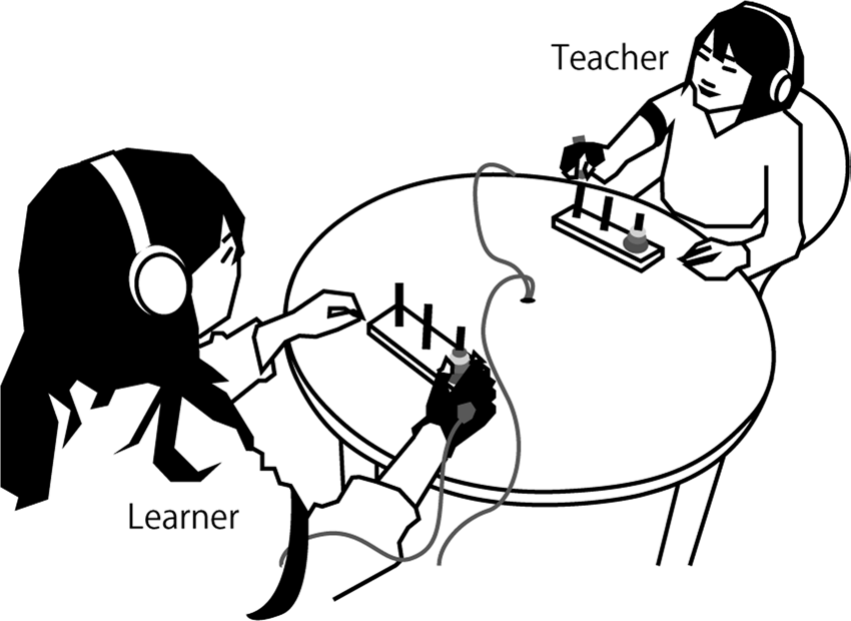
Experimental setup.

In this study, we propose a methodological framework for detecting the interactive scaffolding process in teaching behaviour using an imitation learning paradigm. Through a system identification approach including causality analysis, we investigated the bidirectional information exchange between the motion of teacher and learner during imitation learning. The role of teacher and learner were randomly assigned within each pair of participants. The teacher first solved a wooden puzzle (Tower of Hanoi, Fig. 1) by her/himself to memorize its solution (teacher phase, or TP), and then manually demonstrated the solution to the learner without using any gestures unrelated to the solution or any verbal communication. The learner immediately and simultaneously imitated (shadowed) the teacher’s manual movement (imitation phase, or IP). Finally the learner solved the puzzle alone (learner phase, or LP). We estimated the bivariate autoregressive model for their movements and calculated Akaike causality between them during the IP. In addition, the noise covariance of this model was evaluated to detect the simultaneous component of their movement that was not explained by the bidirectional causality but identified as commonly shared driving information.

**Figure 2:**
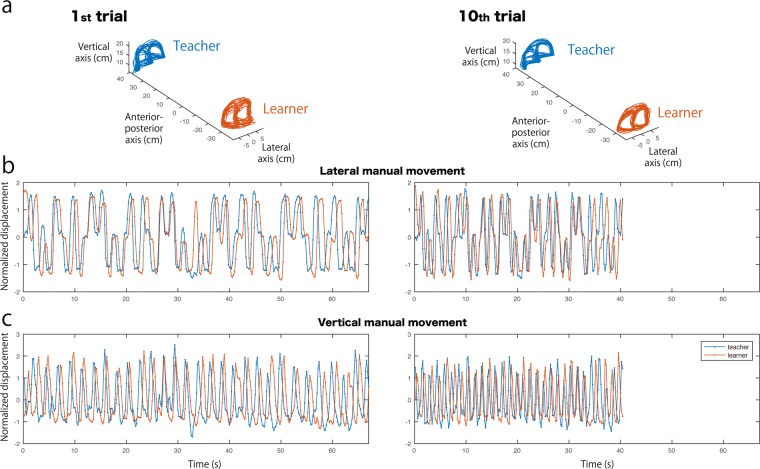
Example of manual movements of teacher and learner (first and tenth trial of a single pair). (**a**) Three-dimensional traces of the manual movements. (**b**) Time series of the lateral motions. (**c**) Time series of the vertical motions. Right and left columns in a–c indicate the manual movements at the first and tenth trials, respectively.

We hypothesized the following bidirectional and commonly shared information transfer between teacher and learner. The first is information transfer from the teacher to the learner indicating the demonstration-imitation relation that communicates and supports the puzzle solution. Second is information transfer from the learner to the teacher indicating the flexible and on-line monitoring process of the teacher for adjusting her/his timing of the demonstration to support the learner’s ongoing progress. Third is information transfer of common procedural knowledge between teacher and learner, allowing them to simultaneously manipulate the puzzle disks, indicating the amount of responsibility of the learner for accomplishing the puzzle without following the teacher’s motion. We expected that these mechanisms would be detected as the causality and the noise covariance, respectively, fulfilling the key characteristics of scaffolding. The support from the teacher to the learner took place contingently to the response from the learner to the teacher. This support faded as the common procedural knowledge was acquired, resulting in the learner solving the puzzle by her/himself. The solution to the Tower of Hanoi is unique, and with four disks the puzzle can be solved in 15 moves from one of the three rods to another (2^4^-1). The lateral component of the manual movements (lateral motion) involves information about the puzzle solution, i.e., the sequence of disk positions (Fig. 2). Thus, the imitation process and knowledge sharing are mainly represented in the lateral motion. In contrast, the vertical component of the manual movements (vertical motion) is rhythmic, and involves timing information for picking and stacking the disk (Fig. 2). Therefore, the teacher’s monitoring process is mainly represented in the vertical motion. This bidirectional and functionally different information transfer and the common driving signal between teacher and learner will provide quantitative evidence of the scaffolding process and the teaching intention in imitation.

## Results

### Behavioural puzzle performance of learners and teachers

**Figure 3:**
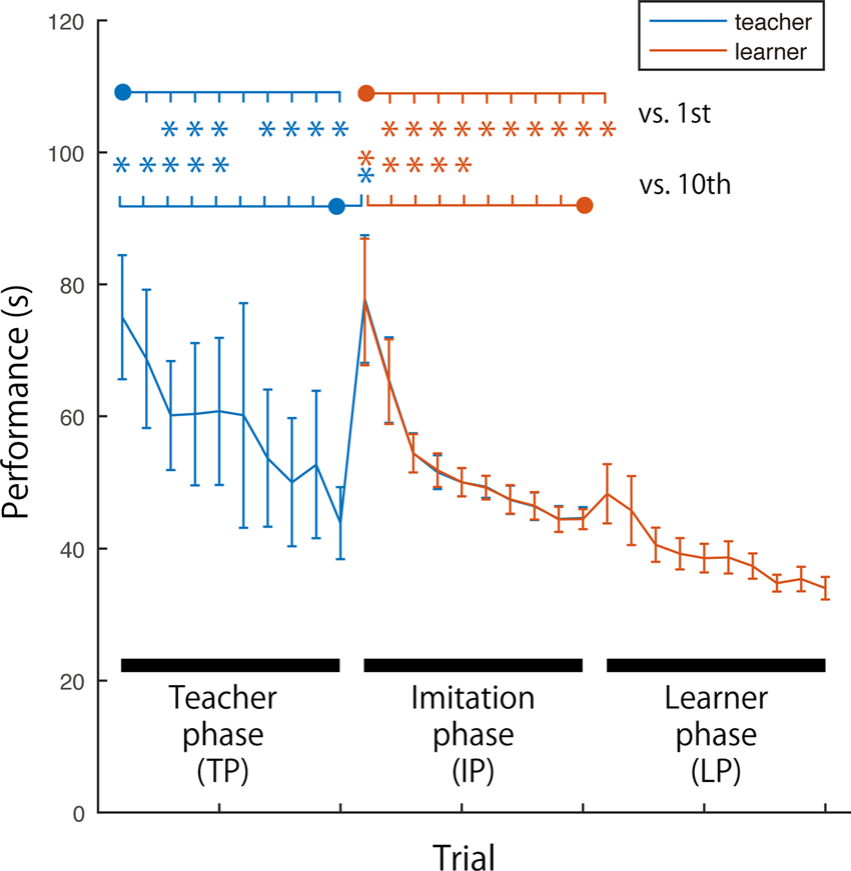
Behavioural results of puzzle solving performance. Blue and orange lines with error bars indicate the duration of solving the puzzle by teacher and learner, respectively. Blue and orange bars with solid circles over the error bars indicate comparison of the durations across trials. The solid circles indicate the reference conditions of these comparisons.

Performance, measured by the time taken to solve the puzzle, was examined across trials (Fig. 3). The puzzle solving duration of the teachers during TP gradually decreased as the learning progressed and the durations of trials 3–5, and trials 7 and later were significantly shorter than for the first trial (3: p = 0.002, R = 0.86; 4: p = 0.008, R = 0.81; 5: p = 0.040, R = 0.73; 7: p = 0.002, R = 0.85; 8: p = 0.001, R = 0.88; 9: p = 0.006, R = 0.83; 10: p = 0.001, R = 0.88). The puzzle duration of learners during IP also gradually decreased with durations of the second trial and later all being significantly shorter than the first trial (2: p = 0.006, R = 0.88; 3: p = 0.001, R = 0.88; 4: p = 0.001, R = 0.85; 5: p = 0.004, R = 0.81; 6: p = 0.009, R = 0.88; 7: p = 0.001, R = 0.88; 8: p = 0.001, R = 0.88; 9: p = 0.001, R = 0.83; 10: p = 0.001, R = 0.88). The performance of learners in the IP significantly improved from the second trial, while that of teachers in the TP improved from the third. Although indirect, the difference in the performance change suggests that imitation learning is more efficient than the solo learning despite the solo learner (teacher) being given written instructions of puzzle solution before the manual learning session began. The puzzle duration of the learners at the first trial of the LP was significantly shorter than the first trial in the IP (p = 0.023, R = 0.76). This indicated that imitation functioned as learning rather than simply mirroring (i.e., doing the same action but not learning). In addition, the puzzle performance was compared with that of the last trials (10^th^ trial). For both teachers in the TP and learners in the IP, the duration of trials 1–5 was significantly longer than that of the 10^th^ trial, resulting in learning reaching an asymptote during the last 5 trials (Teacher in TP: 1:p = 0.001, R = 0.88; 2:p = 0.001, R = 0.88; 3:p = 0.001, R = 0.88; 4:p = 0.004, R = 0.85; 5:p = 0.002, R = 0.86; Learner in IP: 1:p = 0.001, R = 0.88; 2:p = 0.001, R = 0.88; 3:p = 0.003, R = 0.85; 4:p = 0.008, R = 0.81; 5:p = 0.008, R = 0.81). Teacher duration for the first trial in the IP was significantly longer than for the last (tenth) trial in the TP (p = 0.001, R = 0.88) and gradually shortened with the learner. This result suggests that the teacher was affected by the presence of the learner.

### Causal influences and noise covariance in the lateral motions

**Figure 4:**
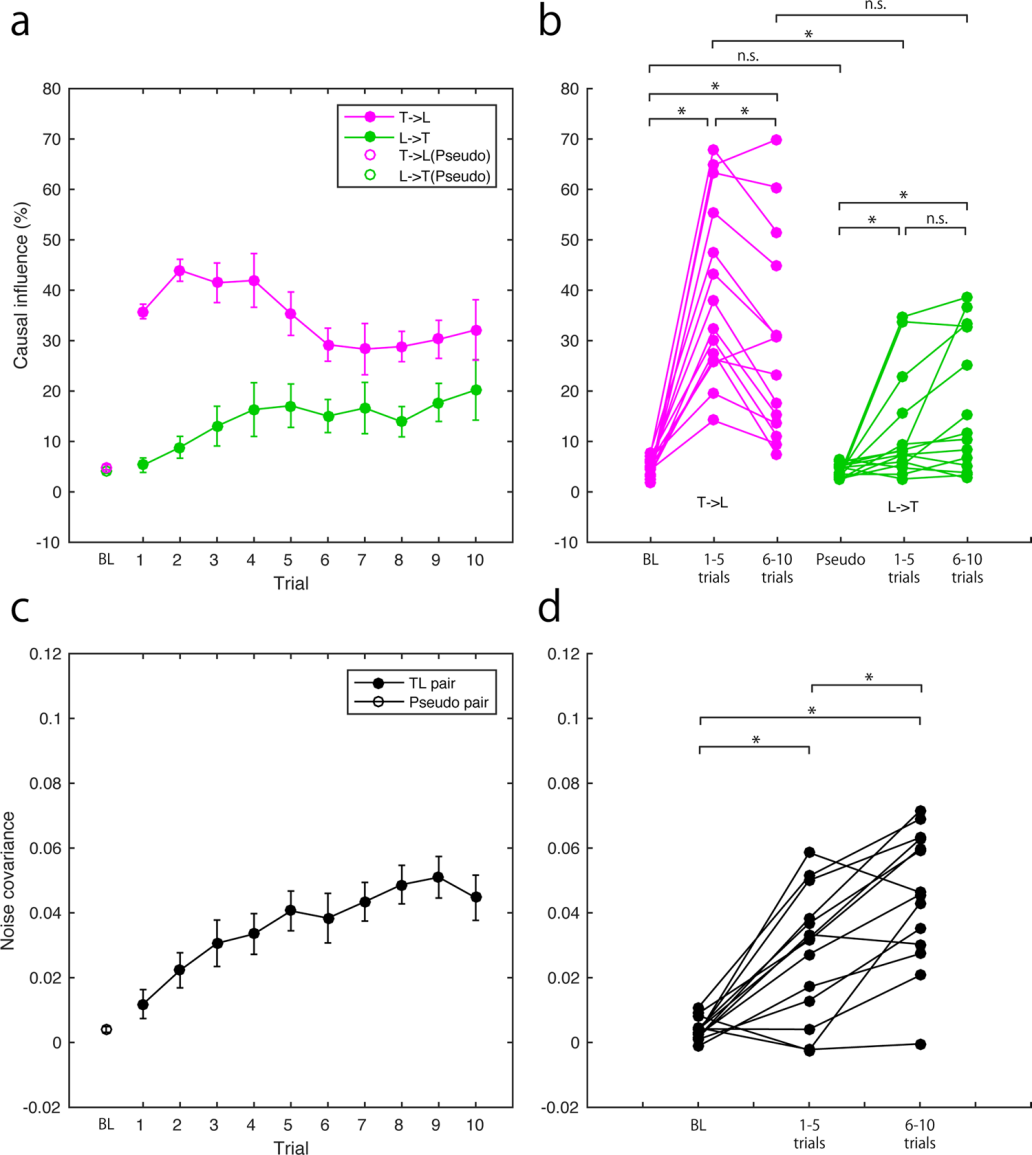
Causal influences and noise covariance in the lateral motions. (**a**) Averaged causal influences between teacher and learner across participants and their progress during imitation (filled circles). Open circles indicate the causal influence calculated for baseline (BL) (see section 4.4). Magenta lines indicate the causal influences from the teacher to the learner. Green lines and markers indicate the causal influences from the learner to the teacher. (**b**) Causal influences of each participant were plotted and compared across conditions (BL, averaged value during first half of trials, and averaged value during second half of trials). Colour of plots and lines corresponds to panel (a). (c) Averaged noise covariance across participants and their progress during imitation (filled circles). Open circles indicate the noise covariance calculated for the BL. (**d**) Noise covariances of each participant were plotted and compared across conditions as in panel (b). Error bars in panels (a,c) indicate standard error of mean. Asterisks in panels (b,d) indicate the significant difference after correction of multiple comparisons by a Bonferroni method (nine repetitions for causal influences; three for noise covariance).

Causal influences between the lateral motions of teacher and learner were calculated during IP. The causal influences from teacher to learner gradually decreased, while those from learner to teacher gradually increased (Fig. 4a). We compared causal influences averaged across the first half of the trials, those averaged across the second half of trials, and those calculated for pseudo pairs and averaged across all trials as baseline condition (BL) (Fig. 4b). Results showed that the causal influence from the teacher to the learner was significantly greater than that of the BL throughout the IP (first half of trials: p = 0.001, R = 0.88; second half of trials: p = 0.001, R = 0.88). The causal influence from the learner to the teacher was also significantly larger than that of the BL throughout the IP (first half of trials: p = 0.036, R = 0.73; second half of trials: p < 0.015, R = 0.78). The causal influence from the teacher to the learner was significantly smaller in the second half of the trials than for the first half (p = 0.036, R = 0.73). Further, the causal influence from the teacher to the learner was significantly greater than the influence from the learner to the teacher during the first half of the trials (p = 0.021, R = 0.76), but the significance disappeared for the second half (p > 1, R = 0.41).

We then evaluated the noise covariance of teacher and learner lateral manual motions. The noise covariance gradually increased (Fig. 4c). The averaged noise covariance for the first and second half trials was significantly larger than that of the BL (first half trials: p = 0.005, R = 0.78; second half trials: p < 0.001, R = 0.86) (Fig. 4d). The comparison of the noise covariance between the first and second half of the trials showed a significant difference (p = 0.005, R = 0.78) (Fig. 4d).

### Causal influences and noise covariance in the vertical motions

**Figure 5:**
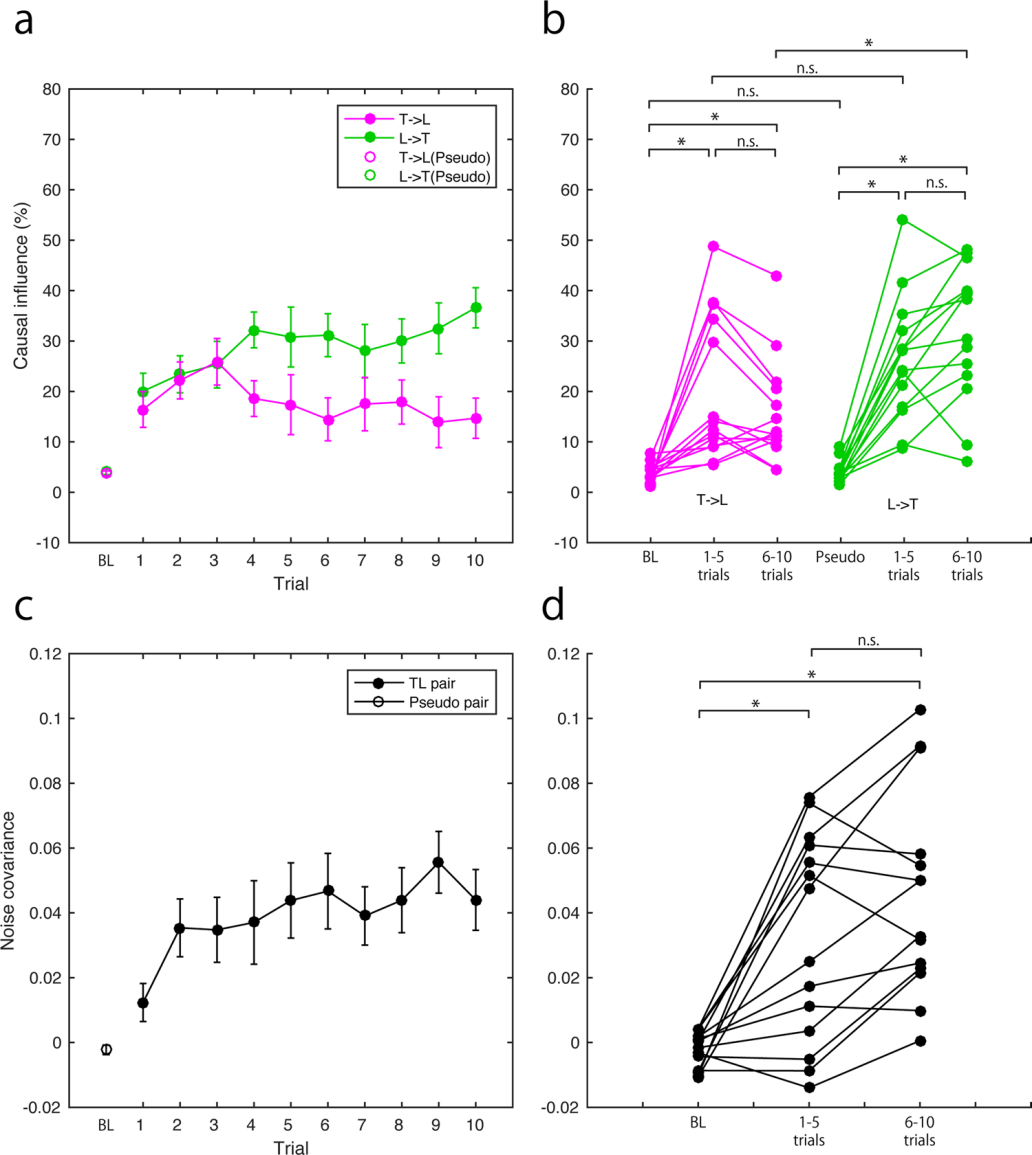
Causal influences and noise covariance in the vertical motions. (**a**) Averaged causal influences between teacher and learner across participants and their progress during imitation (filled circles). Open circles indicate the causal influence calculated for baseline (BL) (see section 4.4). Magenta lines indicate the causal influences from the teacher to the learner. Green lines and markers indicate the causal influences from the learner to the teacher. (**b**) Causal influences of each participant were plotted and compared across conditions (BL, averaged value during first half of trials, and averaged value during second half of trials). Colour of plots and lines corresponds to panel (a). (**c**) Averaged noise covariance across participants and their progress during imitation (filled circles). Open circles indicate the noise covariance calculated for the BL. (**d**) Noise covariances of each participant were plotted and compared across conditions as in panel (b). Error bars in panels (a,c) indicate standard error of mean. Asterisks in panels (b,d) indicate the significant difference after correction of multiple comparisons by a Bonferroni method (nine repetitions for causal influences; three for noise covariance).

Causal influences between the vertical motions of the teacher and learner were calculated during IP. Influence from learner to teacher gradually increased, while the teacher’s influence on the learner gradually decreased (Fig. 5a). The causal influences in the first and second half of the trials were computed and compared, together with comparison with the causal influence of the BL, as for the lateral motion (Fig. 5b). Results showed that the causal influence from learner to teacher, and of teacher on learner, were both significantly larger than that of the BL throughout the IP (teacher on learner, first half trials: p = 0.001, R = 0.88; second half trials: p = 0.002, R = 0.86; learner on teacher, first half trials: p = 0.001, R = 0.88; second half trials: p = 0.001, R = 0.88). In addition, the causal influence from learner to teacher was significantly greater than for teacher on learner for the second half (p = 0.021, R = 0.75).

We then evaluated the noise covariance between the vertical motions. Noise covariance increased across trials (Fig. 5c) and the averaged noise covariance for the first and second half of trials was significantly larger than that at BL (first half trials: p = 0.009, R = 0.75; second half trials: p < 0.001, R = 0.88) (Fig. 5d). However, the comparison of the noise covariance between the first and second half of trials did not show a significant difference between them (p = 0.106, R = 0.56) (Fig. 5d).

### Impulse responses corresponding to causal influences

**Figure 6:**
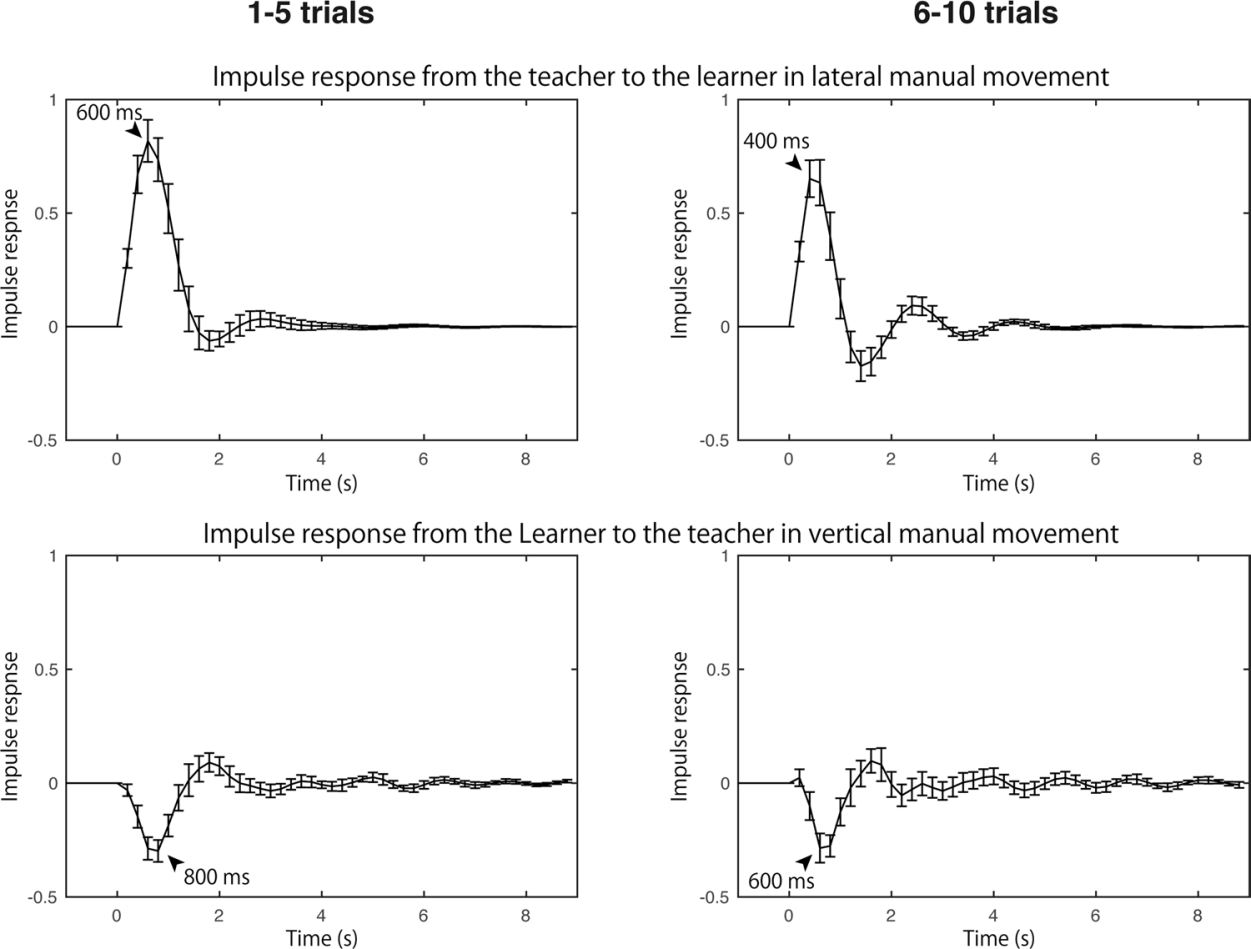
Impulse response corresponding to the causal influences averaged across participants. Superimposed arrows indicate the peak latency (ms). Error bars indicate standard error of mean.

The causal influence from the teacher’s lateral motion to the learner’s was greater than that of the learner to the teacher. In contrast the causal influence of the vertical motion from the learner to the teacher was higher than that from the teacher to the learner. Since the causal influences indicate the amount of influence but not the polarity of the effect, impulse responses corresponding to the dominant causal influence were computed and evaluated (Fig. 6). The impulse response from the teacher to the learner in the lateral motion (i.e. simulated learner’s response where the instantaneous input that height is 1 fed into the teacher on the estimated bivariate autoregressive model) showed a high positive peak with a latency of 400–600 ms (upper panels of Fig. 6). While the impulse response from the learner to the teacher in the vertical motion (i.e. simulated teacher’s response where the instantaneous input that height is 1 fed into the learner on the estimated bivariate autoregressive model) showed a low negative peak with a latency of 600–800 ms (lower panels of Fig. 6).

### Causal influence and noise covariance associated with learner’s behavioural performance

**Figure 7:**
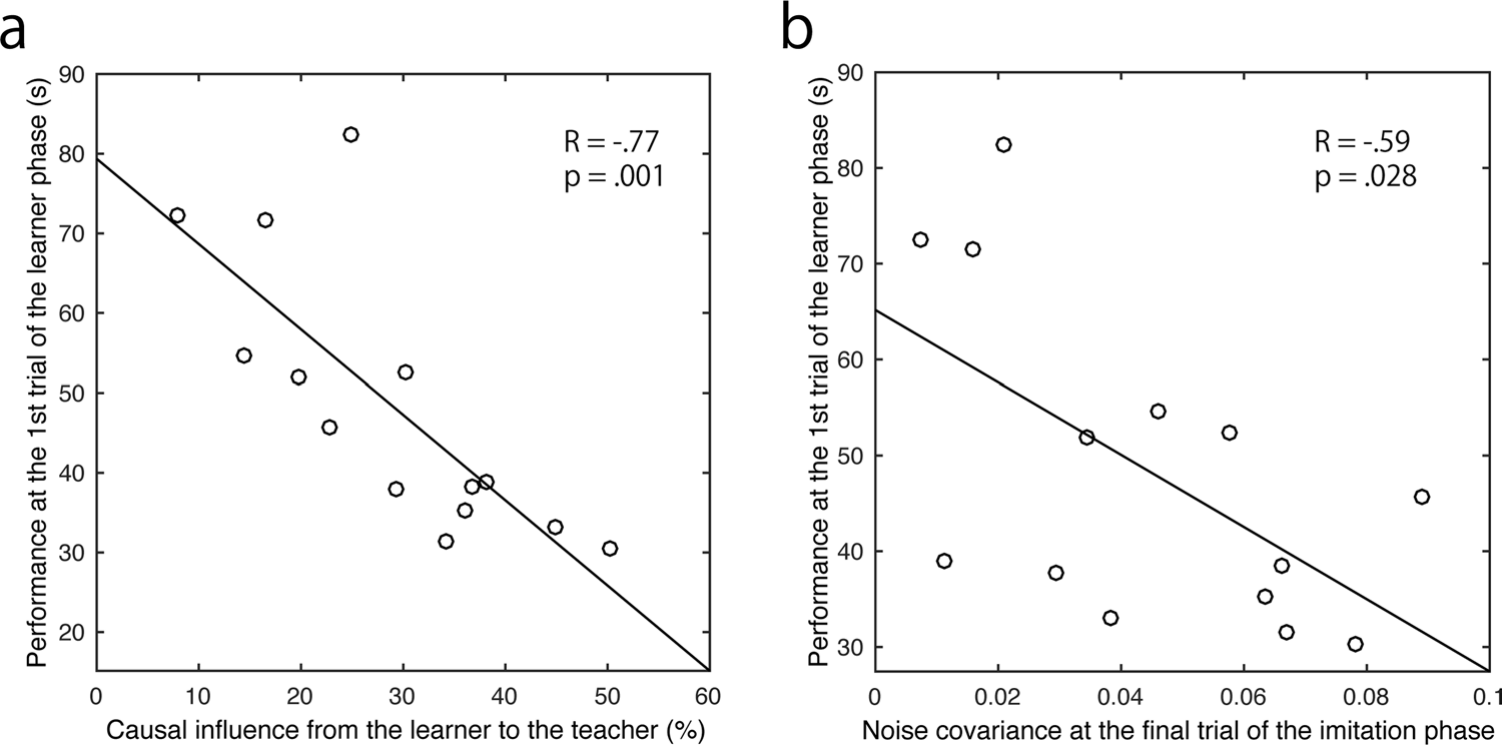
Association between causal influence and noise covariance, and learning performance of the learners at the first trial of the LP.

Finally, we examined the relation between the calculated variables (causal influences and noise covariance) and behavioural performance. We considered that the influence from the learner to the teacher in vertical motions (through IP) indicated the flexible and on-line monitoring process of the teacher for adjusting her/his timing of the demonstration based on the learner’s ongoing progress. We surmised that noise covariance of the lateral motions (at the last trial in IP), indicated shared knowledge and predicted learner performance. As expected, the variables were significantly correlated with the time taken to solve the puzzle at the first trial in the learner phase (LP) (noise covariance: R = −0.59, p = 0.028; causal influence: R = −0.77, p = 0.001) (Fig. 7).

## Discussion

In this paper, we addressed the question of how teaching intention can be identified from the teaching behaviour itself. Based on the assumption that the scaffolding behaviour to promote imitation learning reflects the teaching intention, we hypothesized that quantifying reciprocal interaction among teacher and learner (bidirectional information transfer and sharing common knowledge) allows us to identify the three characteristics of scaffolding, i.e. *contingency*, *fading* and *transfer of responsibility*. There were three main findings in the current study. First, we quantified the bidirectional influences from the manual coordination of teacher and learner in teaching *via* imitation. Second, considering that the knowledge gap between teacher and learner is closed as learning progresses, we quantified the common knowledge that enables one to predict the next move and simultaneously act with a partner during imitation learning. Third, these bidirectional influences and common knowledge quantitatively verify the scaffolding process. We now discuss the functional implications, limitations, and future directions of this work.

Simultaneously recorded manual movement of teachers and learners during imitation allowed us to quantify information exchange in the teaching coordination. Using a bivariate autoregressive model and further causal analysis, we demonstrated existence of the bidirectional causal influences between teacher and learner. Recent studies have argued that teaching should be regarded as a reciprocal interpersonal coordination^1,3,4,24^. This concept has also been empirically verified in computer-human teaching interaction^22^. Our findings are consistent with these previously reported conceptual and computational frameworks of teaching interaction. Note that the causal influence indicates the magnitude and direction of information transfer, but two interpretations that are not mutually exclusive are possible for the changes in the magnitude of causal influences: Magnitude of causal influence can be modified by its transmitter (from whom), its perceiver (to whom), or both. Our previous study^25^ reported that visuo-postural influence from person A to person B decreased when person B closed her/his eyes, but not when person A did this. That is, magnitude of causal influence was mainly modified by the perceiver’s condition. In contrast, D’ausilio and colleagues reported that the magnitude of causal influence from conductor to musician was altered depending on the conductor^26^. These authors, at the same time, noted that: “The musician has to wisely balance several external sources of information and mix them up in order to reach the required performance”, suggesting that it can also be altered by musicians. Because we found the bidirectional causal influences between teacher and learner had different characteristics (form of the impulse response) for each direction (from the teacher to the learner or from the learner to the teacher), the meaning and functions of these bidirectional causal influences were interpreted as follows.

The causal influence from the teacher to the learner is more prominent than that from the learner to the teacher in the lateral component of their movement, i.e. the motion of solving puzzle sequence (Figs 2b, 4). As the impulse response of this causal influence showed a positive peak (Fig. 6), the influence was interpreted as an information transfer of puzzle sequence from demonstrating teacher to the imitating learner. This causal influence significantly decreased as the learning progressed (Fig. 4). There are two explanations for these results. One is that the teacher reduced the help in the demonstration. The second is that the learner became less dependent on the teacher’s demonstration in imitation.

While, causal influence from learner to teacher is more prominent in the vertical component of their movement, i.e. the motion of picking and stacking puzzle disks (Figs 2c, 5). The impulse response of this causal influence showed a negative peak (Fig. 6). This result indicated that the teacher coordinated her/his motion in the opposite direction against the learner’s preceding motion. Given that the vertical motion mainly represents the timing of picking and stacking the disks of the puzzle and showed cyclical features (Fig. 2c), it is not surprising that the teacher behaved in accordance with the learner’s action even if the puzzle sequence of the teacher still preceded that of the learner. Taken together, the causal influence of the vertical motion suggested that the stacking of the teacher followed the learner picking up the same disk, or the picking up of the teacher followed the stacking of the learner for the previous disk. Because this information transfer significantly correlated with the learnt puzzle performance (Fig. 7a), there are two possible interpretations for the causal influence. One is that the teacher who is sensitive to the learner’s progress (advancement or bewilderment) can facilitate the learner’s performance. The second is that the learner, with a clear requirement for help easily perceived by the teacher, learned better. In sum, to our knowledge, this is the first report that there is a bidirectional but functionally different influence in teacher-learner interaction, and in more general interpersonal interaction as well.

We also demonstrated direct evidence of an internal common drive in interpersonal interaction^27,28^. Teaching is a process to close the knowledge gap between teacher and learner^29,30^ who will ultimately both share common knowledge. In this regard, the teacher-learner interaction provides a valuable opportunity to verify the common drive in the interpersonal interaction. We found that the noise covariance of the manual movements of the teacher and the learner increased as the learning progressed. Indeed, the noise covariance during the second half trials was significantly larger than during the first half in the lateral motion (Fig. 4c). Noise covariance is a simultaneous component of teachers’ and learners’ manual movements and cannot be explained by the bidirectional interaction in the estimated model. This suggests that learners could anticipate the timing of their teacher’s motion^31^ and simultaneously moved without merely imitating the teacher. Thus, the level of noise covariance indicates the acquired knowledge of the puzzle solution. This was also supported by the finding that the noise covariance in the lateral motion predicted the initial LP performance (Fig. 7b). In the vertical motion, there was no significant difference between the first and second half of the trials. This is because the vertical motion mainly represents the timing information rather than the puzzle solution.

Summarizing the discussion above, the significant decrease of information transfer from teacher to learner in lateral motion demonstrates the fading of support, one of the key characteristics of scaffolding^18,32^. We also found information transfer from learners to teachers with a negative impulse response, predominant in the vertical motion. This indicates that teachers were waiting for learners to accomplish their picking and stacking before demonstrating the next action. Taken together with the description of teacher support, this suggests empirical evidence of contingency in the scaffolding^18,32^. The extent of the causal influence correlated with the puzzle solving performance by the learners alone. This suggests that the information transfer from learner to teacher reflects the contingent scaffolding, resulting in efficient learning. Given that the noise covariance was a commonly driving signal for their lateral motions to accomplish the puzzle, the increasing noise covariance illustrated that learners began completing the puzzle by themselves as they obtained the knowledge of how to solve it. This is quantitative evidence of transfer of responsibility through scaffolding^18,32^.

Findings in the current study clearly illustrated that the teacher-learner interaction consists of a three-way information transfer: imitation, monitoring the learner’s state, and acquired knowledge (puzzle solution) as a common signal. The behavioural and neural mechanisms of imitation have been thoroughly investigated and the mirror neuron system (MNS) in the brain plays a critical role in this^33–36^. Imitation learning implies learning a novel motor pattern or sequence^37,38^ and requires the MNS as a core region^39–41^. However, processes of monitoring the learner’s state and their common knowledge have been less investigated. We assume that these processes have to be investigated in the context where the learner and demonstrator both actively engage in imitation learning. This assumption is supported by a recent proposal that social interaction involves flexible online adjustments between two agents and cannot be reduced into two individual responses to the interacting partner explored in isolation^42–45^.

In line with these studies, investigations addressing teacher-learner interaction have increased^23,46–49^, although they have not yet fully identified the scaffolding process. Pan *et al*.^23^ reported that brain activities recorded from the inferior frontal cortex (IFC) by near infrared spectroscopy (NIRS) during imitation learning of a song showed interpersonal synchronization which predicted a learner’s performance. These findings can be interpreted as information transfer related to syntactic and/or motor representation of the song from the teacher to the learner because the synchronized activities also showed higher causal influence from the teacher’s IFC to the learner’s IFC than the reverse when the learner observed the instructor’s modelling. It is plausible that this causal influence is compatible with information transfer from the teacher to the learner as we found. The IFC region involves social memory acquired through joint attention^50^. We thus consider that this result can also be interpreted as commonly acquired knowledge or skills represented in the IFC. It is consistent with our result that acquiring common knowledge or skills was associated with the learning performance (Fig. 7b). We also reported that the information transfer from teacher to learner decreased as learning progressed while common knowledge or skills increased as learning proceeded (Fig. 4). Future studies investigating the association between the dynamics of interpersonal brain synchronization in the learning progress are necessary for further clarification of the IFC function.

During a video game teaching-learning task, Takeuchi and colleagues reported that the teacher’s left prefrontal NIRS activity predicts the gap in the teacher’s assessment between quality of teaching and that of learning^48^. These authors also reported that this activity changed in synchrony with the activity of the same region in the student brain. This process is compatible with our proposed information transfer involved in teachers’ monitoring of the learning state. The neural substrate of this process was assumed to be the dorsolateral prefrontal cortex (DLPFC)^48^. It has been previously proposed that the DLPFC engages in the function of selecting and combining the existing, elementary motor representation for imitation learning^35,41^. Taken together, the DLPFC of the teacher, particularly left DLPFC, may also play a role in evaluating learners’ immaturely combined motor elements and selecting appropriate demonstrations to facilitate their learning. Finally, our proposed methodology and quantitative findings of teacher-learner interaction will be helpful for investigating neural mechanisms of imitation learning and teaching in the future.

In the current study, the teacher was instructed to “encourage the learner to solve the puzzle by her/himself as rapidly and accurately as possible on the first trial of the LP”. The teacher was not explicitly instructed to delay the next demonstration until the learner had completed the disk manipulation in each step. The observed flexible timing adjustment of the demonstration, i.e., scaffolding, would have been adopted by the teachers as one of the strategies to promote learning. This timing adjustment may reflect that teachers consider not only the speed of the movements but also if the learner memorizes the sequence. Indeed, the degree of the teacher’s adjustment was associated with the solo performance of the learner (LP) (Fig. 7a). These findings suggest that learners could memorize the sequence of the puzzle solution in parallel with shadowing the demonstrated model. In other words, the contingent demonstration responding to the learner’s need in the memorizing process allowed the teacher to efficiently transfer knowledge. This scaffolding behaviour is consistent with definitions of teaching such as:

“Teaching is bidirectional and consists of a source of knowledge (the teacher), a recipient of that same knowledge (the learner) and the process and mechanisms of transmission of that knowledge when both the teacher and learner actively communicate their understandings to each other^1^”.

or through which a knowledgeable individual “alter[s] his or her behaviour in such a way as to actively help another to learn what the knowledgeable individual knows^7^”. Such contingent scaffolding processes are basically identified as “intentional” tutoring^17^. We found that the teacher’s performance in the IP progressed closely together with the learner’s (Fig. 3). This can be explained by a flexible adjustment of the teacher’s demonstration depending on the learner’s performance. Previous studies have reported a similar scaffolding process when an adult demonstrated the action to infant and robot imitators^19–21^. Such scaffolding process still possibly implied the tutor’s *a priori* assumption that the learner is not equipotent. In our study, it was also possible that a teacher slowed her/his motion enough to be followed by the learner based on *a priori* information, such as the performance when the teacher learned in isolation (TP). If this is the case, however, similar progress of the teacher’s performance in the TP and IP is unlikely to occur because the teacher would take the variation in the learner’s ability into account to achieve their joint progress during the IP. Fukuyama and colleagues have described how a mother’s demonstration to her infant of 11 to 13 months of age was modified depending on the preceding infant achievement, but not for infants of 6 to 8 months of age^20^. This finding suggests that there is flexible adjustment of demonstrating behaviour for imitation. In sum, these previous studies support our assumption.

In this study we sought to operationally define teaching intention through the teacher’s scaffolding behaviour. The major limitation is that the proposed behavioural data analysis cannot discriminate between the scaffolding behaviour caused by the instructed teaching intention from merely helping the learner’s shadowing/imitating behaviour without considering that the learner is trying to learn. The operational definition requires that scaffolding behaviour inevitably involves teaching intention. It should thus be carefully considered in future studies whether or not such scaffolding behaviour fulfills the sufficient condition of teaching intention. Another limitation of this study is the extent to which our methodological framework can be applied. First, the Tower of Hanoi may be difficult for infants and non-human animals^51^. Easier tasks should be adopted for such cases. Second, analysis of this study required two distinct time series to represent spatial and temporal information. The Tower of Hanoi allows us to divide motion into lateral and vertical motion for each of these. When this puzzle cannot be used, appropriate task selection and data extraction will be necessary. Finally, the analysis would not be suitable when demonstration (or observation) and imitation (or execution) occur successively and alternately rather than simultaneously during imitation. If this is the case, the movement to be learnt should at least be segmented into short subcomponents to be demonstrated and imitated (‘part learning^23^’). It is also questionable whether the imitation strategy adopted in this study (shadowing or a rapid imitation^41^) could be achieved by animals, even, for example, by non-human apes. Thus, the suitable behavioural coordination of animals in either the field or naturalistic circumstances in captivity should be addressed. The social facilitation or motor contagion that contribute to social learning of foraging and anti-predator behaviour in animals^52^ represent nearly simultaneous coordination among conspecifics and are straightforward subjects for future studies. Our methodological framework will give an opportunity to investigate whether teaching behaviour can be identified in such primitive social learning situations.

Although there has been much debate about whether the teaching-like behaviour in non-human animals can be defined as teaching^1,5–9,13–15,29^, we consider that our experimental and analytical procedures are useful for identifying teaching and elucidating its mechanisms in non-human animals as well as humans. If animals coordinate with conspecifics and show scaffolding behaviour, it can be termed teaching. This study provides a feasible methodological framework for understanding the general principle of teacher-learner interaction in humans, as well as for non-human animals and robots.

## Methods

### Participants

The participants were 14 pairs of the same sex (8 female and 6 male pairs; age: 20.9 ± 1.2 years). All participants had normal or corrected-to-normal vision, and reported no history of physical, psychiatric or neurological disorders and reported naïve to the task of this study (Tower of Hanoi). Both right- and left- handers participated (one left-handed teacher and two left-handed learners). This study was conducted according to the principles in the Declaration of Helsinki and approved by the academic research ethical review committee of Waseda University, Japan. All participants provided written informed consent and were paid for participation. The data that support the findings of this study are available from the corresponding author upon request.

### Apparatus

The pair of participants sat on acrylic chairs and faced each other across an acrylic round table (Radius: 45 cm). Two wooden puzzles consisting of a stand with three upright rods and four disks pierced with a rod (Hanoi’s Tower) were set on the table and immobilized by Velcro tapes (Fig. 1). Six-dimensional (X, Y, and Z positions, and yaw, roll, and pitch rotation angles) manual movements of the participants were recorded by a three-dimensional magnetic field digitizer (Model 3SF0002, Polhemus, Navigation Science Division, Kaiser Aerospace, VT, USA) and a connected laptop (Let’s Note, Panasonic, Osaka, Japan). The digitizer consisted of a magnetic field transmitter and two receivers. The transmitter was attached beneath the table and aligned so that the X, Y and Z axes of the recording field corresponded to lateral, anterior-posterior, and vertical axes of the participants. Participants wore a fabric glove on their right hand with the thumb, index, and middle fingers uncovered and exposed. Receivers were attached to the gloves (back of the hand) of each participant with Velcro tapes. Cables connected to the receivers were loosely attached to the upper arm with a Velcro band so that they did not interfere with the manual movement. During the experiment, participants wore earplugs and headphones (HD25 SP; Sennheiser Electronic GmbH & Co. KG, Wedemark, Germany) to reduce environmental noise, such as the clacking sound of the puzzle disks.

### Procedure

The duration of the experiment, including instructions and debriefing, took less than 1.5 hours. The roles of teacher and learner were randomly assigned within each pair of participants. The main experiment consisted of three phases. First, one of the pair of participants (teacher) see the written solution of the puzzle for 5 min. Then, in the teacher phase (TP), the teacher performed the puzzle alone so that s/he can solve it as rapidly and accurately as possible. Second, the imitation phase (IP), involved the teacher demonstrating an example of the puzzle solution to the other participant (learner), and the learner immediately and simultaneously imitating and learning it. Finally, in the learner phase (LP), the learner continued to learn how to solve the puzzle by her/himself as rapidly and accurately as possible. Manipulating the four disks from the left pole to the right pole, and then from the right pole to the left pole (30 moves) using the right hand was regarded as one trial for the teacher, and the mirrored manipulation for the learner. All participants were instructed to use only their right hand to manipulate the puzzle disks and to comfortably rest their left hand on the table during the experiment. The three experimental phases consisted of ten trials each. If necessary, additional trials were conducted after the TP until the teachers were confident in perfectly memorizing and demonstrating the puzzle sequence for the learner (one right-handed teacher needed eight additional trials and one left-handed teacher needed one additional trial until they were confident, finally resulting in sufficient performance of the puzzle for all teachers (Mean ± SEM: 39.5, ±1.9 s), see Supplementary Information). In the IP, the teacher was further instructed not to vocalize or use intentional cues not needed for solving the puzzle. The learner was instructed not to wait for the accomplishment of the teacher’s manipulation, but to shadow the movement. Although speed and accuracy in the IP was not explicitly instructed, the teacher was asked to encourage the learner to solve the puzzle by her/himself as rapidly and accurately as possible on the first trial of the LP. Participants’ manual movements were recorded by the magnetic field digitizer with a sampling rate of 120 Hz (60 Hz for each participant).

### Analysis

The simultaneously recorded manual movements of the teacher and learner were segmented from the beginning to the end of puzzle solving based on the velocity of their vertical movement. The duration of the puzzle solution was compared across trials and experimental phases using a Wilcoxon signed rank test. The time series of the lateral and vertical motions, except for the first and last second, were down sampled from 60 Hz to 5 Hz and normalized into Z scores (Normalized displacement in Fig. 2), then subjected to further analyses.

Systematically identifying the manual movements of teachers and learners in the IP by a multivariate autoregressive model, we computed and analysed the following two variables (see section 4.5): (1) Causal influence from the teacher to the learner, and *vice versa*, and (2) Noise covariance in the estimated model, as a common driving signal of their motions. For the BL, we also calculated these variables for a pseudo-pair of participants (teacher’s motion in the TP and learner’s motion in the IP). There are two reasons why we calculated the BL variables based on teacher at TP and learner at IP, not between teacher at TP and learner at LP. Firstly, we intended that one of each time series on which the variables were calculated was shared among the normal condition and the BL. Secondly, the time series of a learner’s motion at the LP was too short to align with the teacher’s one for calculating the causality because the learner in the LP had already learnt the puzzle sequence during the IP. The two variables (causal influences and noise covariance) were each compared at baseline (averaged for all 10 trials), at the first half of trials (trials 1–5), and at the second half of trials (trials 6–10) using a Wilcoxon signed rank test. The causal influences from the teacher to the learner and that of learner on teacher were also compared for the first and second half of trials by the same statistical test. Effect sizes were reported as R, that is, correlation coefficients calculated from Z statistics in the approximated Wilcoxon signed rank test (R = Z/√N).

### Multivariate autoregressive modeling and causal influences between variables

A multivariate autoregressive (MVAR) model, specifically, a bi-variate autoregressive (AR) model such as the one used in this study, is a mathematical model of two time series that can be estimated using the linear sum of the history of the two time series data with the Eq. (1).

1 $$\begin{array}{c}{{x}}_{{t}}=\sum _{{k}=1}^{{p}}{{A}}_{{k}}{{x}}_{{t}-{k}}+{{\omega }}_{{t}}\end{array}$$

where

2 $$\begin{array}{c}{{x}}_{{t}}=[\begin{array}{c}{{x}}_{1}({t})\\ {{x}}_{2}({t})\end{array}]\end{array}$$

3 $$\begin{array}{c}{{A}}_{{k}}=[\begin{array}{cc}{{a}}_{11,{k}} & {{a}}_{12,{k}}\\ {{a}}_{21,{k}} & {{a}}_{22,{k}}\end{array}]\end{array}$$

4 $$\begin{array}{c}{{\omega }}_{{t}}=[\begin{array}{c}{{\omega }}_{1}({t})\\ {{\omega }}_{2}({t})\end{array}] \sim {MVN}(0,{Q})\end{array}$$

5 $$\begin{array}{c}{Q}=[\begin{array}{cc}{{q}}_{11} & {{q}}_{12}\\ {{q}}_{21} & {{q}}_{22}\end{array}]\end{array}$$

For the time series of manual movement of the teacher *x*_1_(*t*) and the learner *x*_2_(*t*), AR model coefficient matrix *A*_*k*_, multivariate normal distribution (MVN) noise *ω*_*t*_, and its variance-covariance matrix *Q* were estimated by a household MATLAB program^25^. *t* is a time step of 200 ms because the sampling rate of the targeted time series was 5 Hz. The model AIC (Akaike’s information criterion) was calculated in the range of an AR order (*p*) from 1 to 10. For the next step, the *p* was fixed at 5, and again estimated the model for each trial and participant because averaged AIC continued to decrease until the *p* was 5, but increased at 6. Note that a noise covariance *q*_12_ can be regarded as a common driving signal among these variables (manual movement of teacher and learner), and subjected to further statistical analyses.

The Eq. (1) can be rewritten using the backward shift operator *B*^*k*^ as

6 $$\begin{array}{c}{{x}}_{{t}}=\sum _{{k}=1}^{{p}}{{A}}_{{k}}{{B}}^{{k}}{{x}}_{{t}}+{{\omega }}_{{t}}\end{array}$$

7 $$\begin{array}{c}=\,{(\sum _{{k}=0}^{{p}}{{A}}_{{k}}{{B}}^{{k}})}^{-1}{{\omega }}_{{t}}\end{array}$$

where

8 $$\begin{array}{c}{{A}}_{0}={I}\end{array}$$

Then, spectrum representation of *x*_*t*_, that is *x*( *f* ), was defined as

9 $$\begin{array}{c}x({f})={A}{({f})}^{-1}\omega ({f})\end{array}$$

where

10 $$\begin{array}{c}\omega ({f})=\sum _{{k}=-\infty }^{\infty }{{e}}^{-i2\pi {fk}}({{\omega }}_{{k}})\end{array}$$

11 $$\begin{array}{c}A({f})=\sum _{{k}=-\infty }^{\infty }{{e}}^{-{i}2\pi {fk}}(-{{A}}_{{k}})\end{array}$$

Power spectrum *P*( *f* ) was thus estimated by *Q* and the frequency response function *A*( *f* ) of the AR model.

12 $$\begin{array}{c}P({f})={A}{({f})}^{-1}Q{\{{A}{({f})}^{{\boldsymbol{T}}}\}}^{-1}\end{array}$$

Matrices of *P*_*x*1_ and *P*_*x*2_ are a power spectrum of *x*_1_(*t*) and *x*_2_(*t*), respectively.

13 $$\begin{array}{c}{{P}}_{{\boldsymbol{x}}1}={|{A}{{({f})}^{-1}}_{11}|}^{2}{{q}}_{11}+{|{A}{{({f})}^{-1}}_{12}|}^{2}{{q}}_{22}\end{array}$$

14 $$\begin{array}{c}{{P}}_{{\boldsymbol{x}}2}={|{A}{{({f})}^{-1}}_{21}|}^{2}{{q}}_{11}+{|{A}{{({f})}^{-1}}_{22}|}^{2}{{q}}_{22}\end{array}$$

The Eqs (13, 14) indicate that the power spectra of *x*_1_ and *x*_2_ involve the combination of the subcomponents made by *x*_1_-driven noise variance (*q*_11_), and *x*_2_-driven noise variance (*q*_22_). Thus, the noise contribution ratio (NCR) can be calculated as the contribution of the input noise of the other parameters (e.g., *x*_2_ in the Eq. (15)) in the power spectrum of the target variable (*x*_1_ in the Eq. (15)). These subcomponent values in the spectral density were originally proposed by Akaike^53^ and termed “Akaike causality”.

15 $$\begin{array}{c}{NC}{{R}}_{{x}2\to {x}1}({f})=\frac{{|{A}{{({f})}^{-1}}_{12}|}^{2}{{q}}_{22}}{{{P}}_{{x}1}}\end{array}$$

16 $$\begin{array}{c}{NC}{{R}}_{{x}1\to {x}2}({f})=\frac{{|{A}{{({f})}^{-1}}_{21}|}^{2}{{q}}_{11}}{{{P}}_{{x}2}}\end{array}$$

The total extent of causal influences was computed using a trapezoidal integration of the Akaike causality to obtain its single values as below.

17 $$\begin{array}{c}{Causal}\,{influenc}{{e}}_{{\boldsymbol{x}}2\to {\boldsymbol{x}}1}=\frac{{\int }_{0}^{\frac{{fs}}{2}}{|{A}{{({f})}^{-1}}_{12}|}^{2}{{q}}_{22}{df}}{{\int }_{0}^{\frac{{fs}}{2}}{{P}}_{{x}1}{df}}\end{array}$$

18 $$\begin{array}{c}{Causal}\,{influenc}{{e}}_{{x}1\to {x}2}=\frac{{\int }_{0}^{\frac{{fs}}{2}}{|{A}{{({f})}^{-1}}_{21}|}^{2}{{q}}_{11}{df}}{{\int }_{0}^{\frac{{fs}}{2}}{{P}}_{{x}2}{df}}\end{array}$$

For each lateral and vertical motion, calculated noise covariance and causal influences were compared across trials (first and second half of trials in IP) and with those calculated in the BL using a Wilcoxon signed rank test. Effect sizes were reported as R, as for behavioural performance. In the case where causal influences were significant in relation to those in the BL, impulse responses were calculated and assessed regarding the response polarity to implicate their functions.

Finally, to test our hypothesis, the noise covariance in the last trial in the IP and causal influence from learner to teacher in the lateral motion averaged during IP were subjected to correlation analysis with the learner performance on the first trial of the LP.

## Supplementary information

Teacher-learner interaction quantifies scaffolding behaviour in imitation learning
