# Supplementary material for: Teacher-learner interaction quantifies scaffolding behaviour in imitation learning
Source: Sci Rep. 2019 May 17;9:7543. doi: 10.1038/s41598-019-44049-x (PMC6525160; doi:10.1038/s41598-019-44049-x)
Supplement: Supplementary file 1 — Teacher-learner interaction quantifies scaffolding behaviour in imitation learning [file 41598_2019_44049_MOESM1_ESM.doc]

**Supplementary Information**

**Teacher-learner interaction quantifies scaffolding behaviour in imitation learning**

Shuntaro Okazaki, Yoshihiro Muraoka, and Rieko Osu

Correspondence to: syunta525@gmail.com

**Supplementary Method and Results**

To validate the teacher’s performance before teaching, we acquired additional data from 7 participants (4 female and 3 males; age: 21.3 ± 1.8 years)). They were asked to move the disks of the tower of Hanoi 30 times, which was the same number as that required when actually solving the puzzle in the main experiment, in any order they preferred except manipulating the same disk repeatedly. They took 35.7±1.4 (Mean±SEM) seconds on average to accomplish this free task, which was not different from the teacher’s performance before starting the IP (Mean±SEM: 39.5±1.9 s; CI: 35.4-43.6; see also Figure S1). Thus, the teacher in the current study knew the materials well enough and performed the task smoothly and quickly enough to demonstrate the puzzle sequence to the learner without difficulties.

**
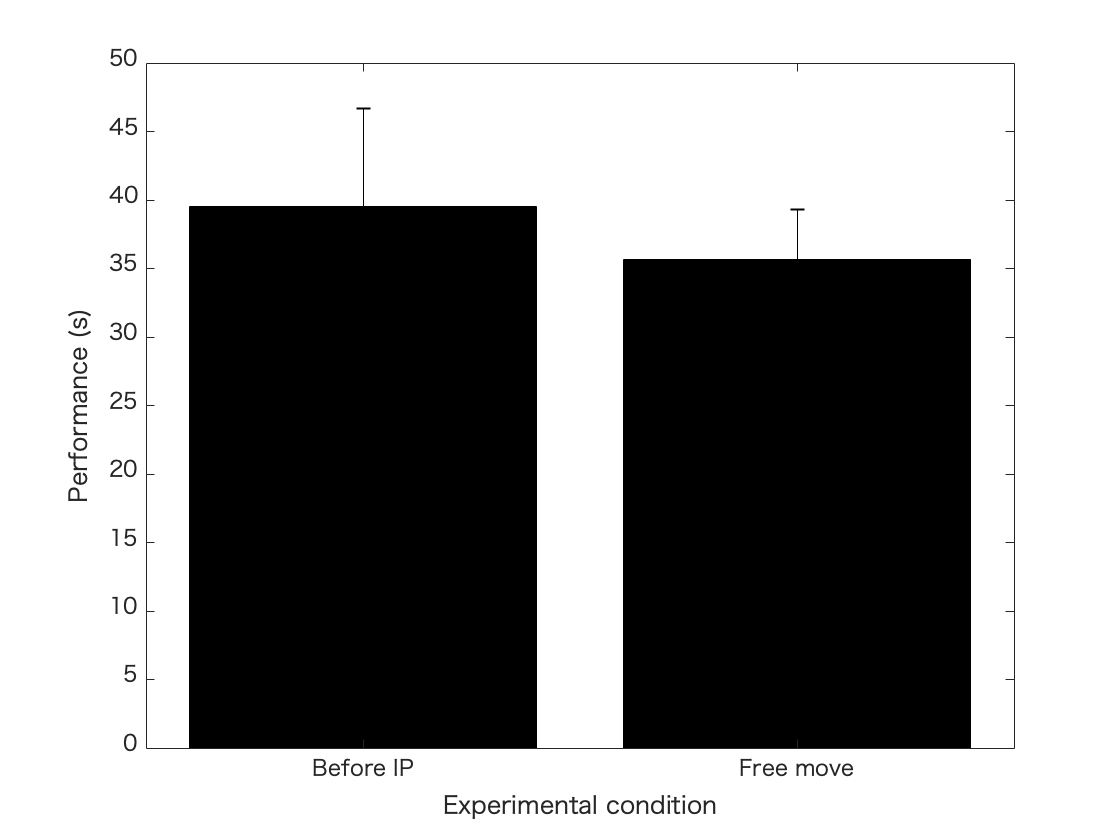
**

**Figure S1. The final performance of the teacher before teaching.**

The puzzle solving duration of teachers before the IP was not significantly different from the performance of 7 additional participants that freely moved the puzzle disks (Mann-Whitney U test, p = .287, R = 0.25). Error bars indicate the standard error of mean.
